# Wood Hemicellulose‐Based Spray‐Dried Microencapsulation of a Lytic Bacteriophage Preserves Phage Viability and Improves Control of the Bacterial Wilt Pathogen *Ralstonia solanacearum*

**DOI:** 10.1111/1751-7915.70315

**Published:** 2026-02-13

**Authors:** Alba M. Negroni, Connor G. Hendrich, Minh Thao Ho, Amin Yousefvand, Ville‐Petri Friman

**Affiliations:** ^1^ Department of Microbiology University of Helsinki Helsinki Finland; ^2^ Department of Food and Nutrition University of Helsinki Helsinki Finland; ^3^ Helsinki Institute of Sustainability Science (HELSUS) University of Helsinki Helsinki Finland; ^4^ HAMK Bio Research Unit Häme University of Applied Sciences Hämeenlinna Finland; ^5^ Oy Medfiles Ltd Vantaa Finland

**Keywords:** bacterial wilt disease, bacteriophage survival, phage encapsulation, *Ralstonia solanacearum*, spray‐dried microencapsulation, wood hemicellulose

## Abstract

*Ralstonia solanacearum* (RS) is a soil‐borne phytopathogen responsible for bacterial wilt disease on a wide range of crops worldwide. Bacteriophage biocontrol is a promising sustainable RS management method. However, more work is needed to design methods to store, ship and apply phage that are effective, scalable and environmentally friendly. Here, we investigate the use of wood hemicellulose excipients—glucuronoxylans (GX) and galactoglucomannans (GGM) – to encapsulate phage PYO4, which can infect the pandemic RS strain UW551. Yield and preservation efficiencies of GX and GGM were compared to the conventional excipient maltodextrin (MD). Encapsulation via spray drying was carried out at two inlet/outlet temperatures, and the resulting powders were stored at room temperature or at 4°C. Phage titers were measured after spray drying, and then weekly for 25 weeks. GX yielded the highest titre of encapsulated phage and preserved phage survival effectively at 4°C. Phages encapsulated with MD had the highest stability at room temperature. GGM had poor results, with low survival after spray drying and low long‐term stability at either temperature. In vitro experiments demonstrated that encapsulated phages inhibited RS as efficiently as unencapsulated phage. Phage encapsulated in GX and MD also reduced bacterial wilt symptoms on tomato. At low MOIs, phage encapsulated in GX and MD reduced symptoms more than unencapsulated phage, suggesting the excipients themselves could be affecting RS. We found that GX alone could inhibit RS growth in vitro and reduce disease progression *in planta* without phage. MD alone couldn't significantly reduce bacterial wilt symptoms or inhibit RS growth in vitro. Together, these results show that the encapsulation of phages in hemicelluloses has great promise for efficient biocontrol methods to combat plant pathogens. Not only are hemicelluloses effective in phage preservation, but also have potential to enhance the biocontrol efficacy of phages through their antimicrobial activities.

## Graphical abstract

Wood hemicelluloses derived from the forestry industry can be used to microencapsulate lytic bacteriophages for storage and application. Phages targeting the global bacterial wilt pathogen *Ralstonia solanacearum* were encapsulated in hemicelluloses that not only preserved them for long term storage but synergistically improved their anti‐*Ralstonia* activity in the soil.

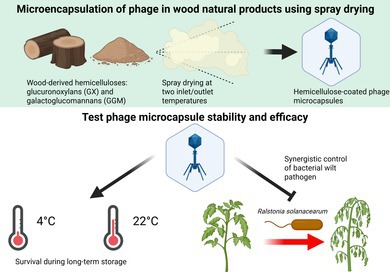

## 1 Introduction

*Ralstonia solanacearum* (RS) is one of the most devastating soil‐borne bacterial phytopathogens globally, causing bacterial wilt disease across economically critical crops including potato, tomato, banana and over 200 other solanaceous and non‐solanaceous species (Vailleau and Genin 2023). RS infiltrates its host through wounds and natural openings in the roots, spreads within xylem vessels and produces extracellular polymeric substances that disrupt water transport, ultimately leading to wilting symptoms (Genin and Denny 2012). The pathogen's remarkable environmental persistence, surviving in water sources for years without virulence loss, and its broad host range pose a significant threat to global food security (Wang et al. 2019). Current European regulations prohibit irrigation of RS‐infected crops and annual losses reach billions of dollars worldwide due to crop destruction and trade restrictions (Álvarez, López, and Biosca 2022). Unfortunately, there is currently no efficient method to control RS once it becomes established in the soil, highlighting the need for novel methods to manage and control this pathogen (Yuliar et al. 2015). The limitations of conventional agrochemical pesticides, coupled with growing regulatory restrictions on chemical pesticides and consumer demand for sustainable agriculture, necessitate innovative, environmentally compatible biocontrol strategies that can effectively manage RS while preserving ecological balance.

Bacteriophages—obligate bacterial parasitic viruses—are promising biocontrol agents that offer host‐specific pathogen control without disrupting beneficial microbiota (Wang et al. 2019; Álvarez and Biosca 2017). Through their lytic lifecycle, phages hijack bacterial cellular machinery to replicate and subsequently lyse host cells, providing self‐amplifying therapeutic action that reduces initial application requirements (Álvarez, López, and Biosca 2022). Recent studies demonstrate successful phage‐mediated RS control in various crop systems, with cocktails of multiple phages showing promise for mitigating resistance evolution while enhancing treatment efficacy (Wang et al. 2019; Álvarez et al. 2019; Álvarez and Biosca 2017). However, critical limitations persist, including phage sensitivity to environmental stressors such as UV radiation, temperature fluctuations and pH variations, which rapidly degrade viral particles and compromise treatment persistence (Malik 2021). These problems are not only relevant when phages are in the environment, but also during the production, shipping and storage of phage formulations. Loss of phage viability prior to application is a significant limit to phage therapy implementation at scale.

While phage particles can be very stable for long periods in liquid suspensions in SM buffer at 4°C, this form of phage storage is inefficient for practical applications as it adds significant additional volume and weight from the buffer itself. As such, there has been great interest in storing phage in a dried state that can be easily shipped and stored. In this context, microencapsulation using a variety of excipients and techniques converts phage solutions into dry powders by creating protective microcapsule matrices that shield phage particles from environmental degradation during processing, storage and field application and facilitate their controlled release upon contact with target environments (Eveliina et al. 2024; Malik et al. 2017). Freeze‐drying (lyophilization) of RS phages using glycerol, sucrose or trehalose as cryoprotectants successfully preserved one phage strain in a stable, dried form, with only a tenfold decrease in phage titre (Álvarez, Gadea‐Pallás, et al. 2022). However, this process did not work as efficiently for all phages tested, with two other phages showing much lower efficiency. Given that whole classes of phages are unsuitable for lyophylization (Huang et al. 2025) and that freeze‐drying can be costly, employing a diverse range of preservation techniques is advantageous. Spray drying represents a promising alternative, as it has been widely used to protect various biological molecules and microorganisms (Yan and Kim 2024). During spray drying, a solution containing the active molecule or microorganism and a solid excipient are first aerosolized and then dried, removing the solvent and producing small particles of excipient containing the active molecule or microorganism (Eveliina et al. 2024; Malik 2021). The process is cost‐effective and easily scalable, making it an attractive technique for industrial applications. This process has been used to encapsulate organic compounds, antibiotics, proteins and even whole probiotic microbes (Tu et al. 2020; Yan and Kim 2024). The technique has also been used with phage (Li et al. 2025; Chang et al. 2019). While the technique varies based on the excipient used, high titers with high stability over periods as long as 4 years have been achieved (Li et al. 2025). However, spray drying is a complex process influenced by multiple factors and must be optimised for the specific conditions of each bioactive compound or phage.

A range of factors must be considered when optimising spray drying conditions. One key factor is the spray drying temperature, which can have significant effects not only on the stability and survival of the material being encapsulated but also can affect the water activity and moisture content of the resulting powder, which can affect the long‐term stability of the encapsulated material (Malik 2021; Ho et al. 2024). The choice of excipient is also crucial, as a variety of monomeric and polymeric molecules are suitable for spray drying applications (Ke et al. 2022). However, even for individual phages, different excipients can have varied effects. One investigation of spray drying using *Pseudomonas aeruginosa* phages found widely varying survival levels based on the level of lactose or trehalose used in the excipient solution (Chang et al. 2019). Drying excipients themselves are also not necessarily neutral and could affect both the target microorganism directly or the surrounding environment. For example, a metareview of the effects of the commonly used spray drying excipient maltodextrin on the human gut microbiome found that, despite its common use as a placebo in clinical trials, maltodextrin often causes a significant shift in microbiome composition and host biology (Almutairi et al. 2022). Given the variation in survival and storage stability of phage within individual drying conditions, expanding our arsenal of spray drying techniques and excipients will increase the repertoire of phage available for agricultural and clinical applications.

Here, we investigate the use of two wood hemicelluloses including hardwood glucuronoxylans (GX) and softwood galactoglucomannans (GGM), as spray drying excipients for an RS‐infecting phage. Phage encapsulation with hemicelluloses through spray drying represents a sustainable and affordable option in the field of bacteriophage biocontrol. GX and GGM are complex mixtures of monomeric and polymeric byproducts of the forestry industry and can be extracted with a process that only employs hot and pressurised water, without the addiction of toxic chemicals. GX and GGM have previously been used to encapsulate plant‐based oils (Ho, Lehtonen, et al. 2023) and probiotic lactobacilli (Eveliina et al. 2024). Their good emulsifying properties, high water solubility, low viscosity, high heat stability and low heat transfer make them attractive spray dying excipients (Ho, Lehtonen, et al. 2023). This study investigates the potential of GX and GMM as excipients for the bacteriophage PYO4, a lytic podophage that can infect the cool‐tolerant RS strain UW551 (Swanson et al. 2005; Franco Ortega et al. 2024). We have previously tested the ability of PYO4 to lyse and control UW551 growth both in vitro and in the rhizosphere and found it produces clear plaques in solid media, inhibits UW551 growth well in liquid media, and is effective at controlling bacterial wilt disease both individually and in combination with other phages (Franco Ortega et al. 2024). We investigated several key aspects of the spray drying process. We spray‐dried phage suspensions at high (170°C/70°C) and low (105°C/50°C) temperatures to refine how spray drying conditions changes the physical characteristics of the spray‐dried powder and the survival of the phages. The physiochemical properties, including water activity, moisture content and particle size, are important as they influence storage stability by governing biochemical degradation of the phage and the overall efficiency of the spray drying process. We then compared the storage stability of phage encapsulated in GX and GGM at room temperature (21°C) and 4°C. We further investigated any potential effects of the complex mixture of potential carbon sources and antimicrobial activity of wood hemicelluloses on RS growth in vitro and in the soil. We finally tested the efficiency of hemicellulose‐encapsulated phages to infect RS both in vitro and in the rhizosphere compared to non‐encapsulated phage and the excipient material alone. In all cases, we used the conventional excipient maltodextrin (MD) as a comparison. We found that GX can serve as an effective excipient and encapsulated phage can inhibit RS more effectively than unencapsulated phage in the soil. Further, we found that GX alone has mild anti‐RS growth properties in vitro and can reduce bacterial wilt symptoms on tomato. GX activity synergistically improved RS growth inhibition in vitro raising the possibility that spray drying excipients could be chosen to enhance phage therapy efficacy and longevity. The results contribute to the development of wood‐derived excipients, offering an eco‐friendly alternative to conventional synthetic polymers with strong protective properties for bacteriophages and support scalable phage therapy applications for agricultural disease management.

## 2 Results

### 2.1 Spray Drying With Wood Hemicelluloses Produced Uniform Particles With Low Water Content

We conducted three independent spray drying runs for the RS podophage PYO4 with two drying temperatures and three excipients (Table 1). To estimate the success of our spray drying conditions, we measured a range of physical characteristics of the produced powders (Figure S1). Overall, the process yields (recovery of solid mass after spray drying) (Tonon et al. 2008; Bhandari et al. 1997) were the highest while using GX as an excipient (Figure S1a). Process yields for GX were between 43.6% and 44.6% across tested temperatures, outperforming both MD, 40.7%–43.7% and GGM, 40.2%–42.4% (Figure S1a). The moisture content of encapsulated phage ranged from 5.05% to 7.57% across all formulations, while water activity values spanned 0.28–0.36. These parameters exhibited temperature‐dependent trends: high drying temperatures reduced both moisture content and water activity compared to low‐temperature processing (Figure S1b,c, *p* < 0.05). For instance, GX microcapsules at high temperatures showed 5.12% moisture content and 0.28 water activity versus 7.57% moisture content and 0.36 water activity at low temperatures. Particle size was consistent across excipient and conditions, with a volume‐weighted mean (D[4,3]) of 6.5–10.4 μm and a surface‐area‐weighted mean (D[3,2]) of 4.7–6.5 μm (Figures S1d and S2e). No statistically significant size differences were observed between any of the treatments, suggesting that neither excipient nor drying temperature significantly altered the final particle size. The observed particle size range is suitable for encapsulation of bacteriophages, as the microcapsules are sufficiently large to accommodate viral particles. Scanning electron microscopy revealed that PYO4 microcapsule powders exhibited predominantly spherical morphology with varying surface characteristics across different excipients and processing conditions (Figure S2). Most particles displayed wrinkled and folded surfaces or appeared relatively smooth, consistent with typical spray‐dried polymer‐based powders (Vehring et al. 2007). The particles generally appeared intact, although some particles could be observed with cracks, ruptures and blow‐holes (Figure S2).

**TABLE 1:** Sample codes of the formulations of different excipients, outlet and inlet air temperatures (H‐high, L‐low) investigated at 20% (w/w) solid feed concentrations in this study.

Sample codes	Excipient	Outlet drying air temperature (°C)	Inlet drying air temperature (°C)
GX‐H	Glucuronoxylans	70	170
GX‐L	Glucuronoxylans	50	105
GGM‐H	Galactoglucomannans	70	170
GGM‐L	Galactoglucomannans	50	105
MD‐H	Maltodextrin	70	170
MD‐L	Maltodextrin	50	105

### 2.2 Drying Temperature Conditions and Excipient Choice Affect Phage Survival During Spray Drying

**FIGURE 1:**
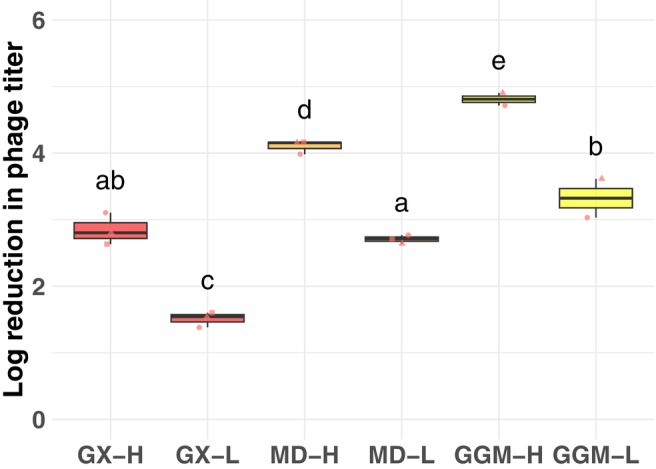
Lower drying temperatures produced higher yields of surviving phage after spray drying. Phage titers were measured before and after spray drying in three excipients and two drying temperatures, 170°C/70°C (H) and 105°C/50°C (L). Data from three spray drying runs are included, with the exception of GGM, where a low starting phage concentration in one replicate led to a phage titre below the level of detection. Phage dried at low temperatures had greater survival than phage dried at high temperatures. GX had the greatest survival and GGM the lowest (one‐way ANOVA, followed by Tukey post hoc analysis, *p* < 0.05, different letters indicate significant differences between treatments).

Phage titers before and after spray drying were measured to calculate the phage survival during the drying process (Figure 1). While all spray dried samples saw a reduction in phage titre over one log, all but two had detectable phage. The third replicate for GGM did not have any detectable phage in either the high or the low spray drying conditions. This replicate started with a lower initial phage titre (~7.8 × 10^7^ PFU/mL rather than ~9.2 × 10^9^ PFU/mL). For GX and MD, the third replicate had a drop in phage titre similar to the other two replicates, suggesting that poorer phage survival was not explained by lower starting concentrations of phage. For GGM, the drop in the third replicate placed the titre below the limit of detection at both high and low temperatures. As we could not confidently estimate the true titre for these samples, we decided to exclude the two samples from further analysis. Both the excipient and the drying temperature significantly affected the survival rate of phage during spray drying (Figure 1). For all excipients, spray drying at the lower temperature had a drop in titre about 1.5 log lower than samples spray dried at the higher temperature. Comparing excipients, GX showed the highest survival, with titre drops of 1.5 log at low temperature and 2.8 log at high temperature. GGM showed the lowest survival, with titre drops of 4.7 log at low temperature and 5.7 log at high temperature. MD had an intermediate phenotype, with titre drops of 2.7 log at low temperature and 4.1 log at high temperature.

### 2.3 Phages Exhibited High Survival at 4°C and Low Survival at Room Temperature

**FIGURE 2:**
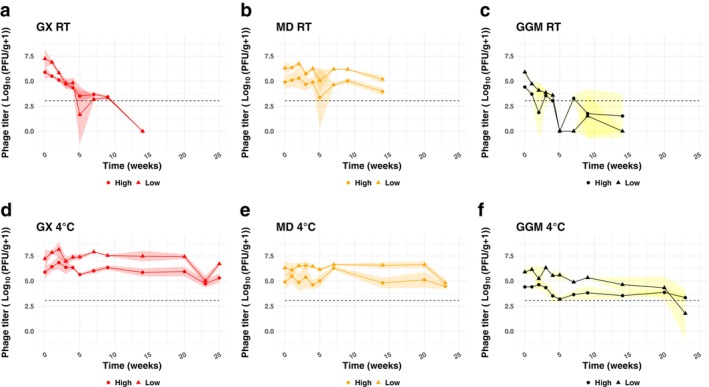
Encapsulated phage were stable at 4°C but had lower stability at room temperature. After spray drying, surviving phage titers were monitored weekly for 14–25 weeks. Phages were encapsulated with: GX (glucuronoxylans) (a, b), MD (maltodextrin) (b, e) and GGM (galactoglucomannans) (c, f) at either high or low spray drying temperatures. The resulting powders were stored at room temperature (RT) (a–c) or 4°C (d–f). Each experiment was conducted in triplicate, with the exception of GGM where one replicate had no detectable phage after spray drying. Dotted lines indicate the limit of detection. Shaded areas indicate the standard deviation.

To determine how the excipient choice and spray drying characteristics affect long‐term phage survival, we observed the phage titers in encapsulated phage powders weekly for 6 months (Figure 2, individual values in Figure S3). Further, we stored the samples at two temperatures, 4°C and room temperature (21°C), to test how storage conditions affect phage survival. Factorial ANOVA analysis of phage survival over the course of the experiment revealed that phage survival was significantly affected by the excipient, storage temperature and the interaction between excipient and storage temperature (Table S1, adj *p* < 0.05). While there was a slight trend towards increased survival in powders dried at high temperatures, perhaps suggesting that the lower moisture content of those powders could play a role in phage long‐term survival, the effect was not significant (Figure 2, Table S1, adj *p* > 0.05). At room temperature, phage titers in GX and GGM dropped steadily, until phages could not be observed in the majority of the samples by week 15, whereas MD encapsulated samples only dropped between 0 and 2 log by week 15 (Figure 2a–c). At room temperature, significant differences were observed between all three excipients, with MD having the highest survival, GX with intermediate survival, and GGM having the lowest survival (Figure S4). Survival was much higher at 4°C, with all three excipients having detectable phage by week 23 (Figure 2d–f). Both GX and MD had significantly higher stability at 4°C than GGM, which exhibited a drop of roughly 4 log for samples dried at low temperature and 1 log for samples dried at high temperature (Figure S4). GX and MD, in contrast, had drops in phage titre between 0 and 2 log at 4°C and were not significantly different from each other. For both GX and GGM, powders stored at 4°C exhibited significantly higher stability than those stored at room temperature (Figure S4). MD encapsulated phage, in contrast, survived relatively well at room temperature, with no significant differences between powders stored at either temperature.

### 2.4 Encapsulated Phages Adsorb and Infect *R. solanacearum* as Well as Unencapsulated Phage In Vitro

**FIGURE 3:**
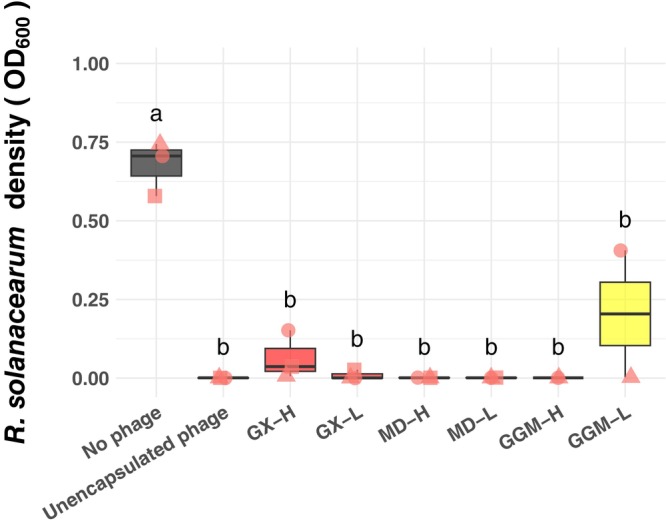
Encapsulated bacteriophages retained their ability to inhibit RS growth in vitro. RS was grown in rich CPG media for 48 h either alone or with phage treatments. Phages, either free or encapsulated, were added to a final MOI of 1. All phage treatments, regardless of encapsulation, encapsulation material, or spray drying temperature, significantly reduced RS growth (one‐way ANOVA, followed by Tukey post hoc analysis, *p* < 0.05, different letters indicate significant differences between treatments).

To determine if encapsulation affects the ability of phage to efficiently infect RS in liquid culture, we challenged RS with encapsulated and unencapsulated phages in rich media (Figure 3). The experiment was conducted 2 months after spray drying and we used powders stored at 4°C for all three excipients, as they had a higher titre than samples stored at room temperature at that time point. All encapsulated phages inhibited RS growth comparably to unencapsulated phages (*p* > 0.05) regardless of the excipient or spray drying temperature used. Six months after spray drying, we tested the adsorption efficiency of encapsulated and unencapsulated phages to assess phage structural integrity (Figure S5). We focused on phage encapsulated at low temperature in GX or MD and stored at 4°C. GGM was excluded from these and further analyses because, at this point, all GGM microcapsule powders had very low levels of surviving phages. All replicates of GX encapsulated phages maintained a high adsorption efficiency compared to unencapsulated phage. More variation in adsorption efficiency was observed in the MD‐encapsulated phage, but there was no significant reduction in adsorption efficiency compared to non‐encapsulated phage (Figure S5). These data suggested that the spray drying did not reduce the phage adsorption or infectivity.

### 2.5 Encapsulation of Phage Provides Protection From UV

One common environmental stressor that can reduce phage titers during storage or in the environment is UV stress (Jones et al. 2008; Ignoffo and Garcia 1994). We tested the ability of GX and MD encapsulation to protect the phage both in powder form and after resuspension in water. As comparison, we used an equal titre of phage suspension in SM buffer. After 30 min of UV treatment, no surviving phage could be detected in samples suspended in SM buffer (Figure S5). In contrast, phage encapsulated in either GX had a significantly lower reduction in phage titre, with GX powder seeing a roughly 20% reduction and GX solution a 30% reduction in phage titre (One‐way ANOVA, *p* < 0.05). MD provided some protection as a powder, with a 55% reduction in phage population. However, this was not significantly different than SM media, and MD did not seem to provide protection after resuspension, with no surviving phage detected after UV treatment (Figure S6).

### 2.6 GX and MD Excipients Alone Affect *R. solanacearum* Growth In Vitro

**FIGURE 4:**
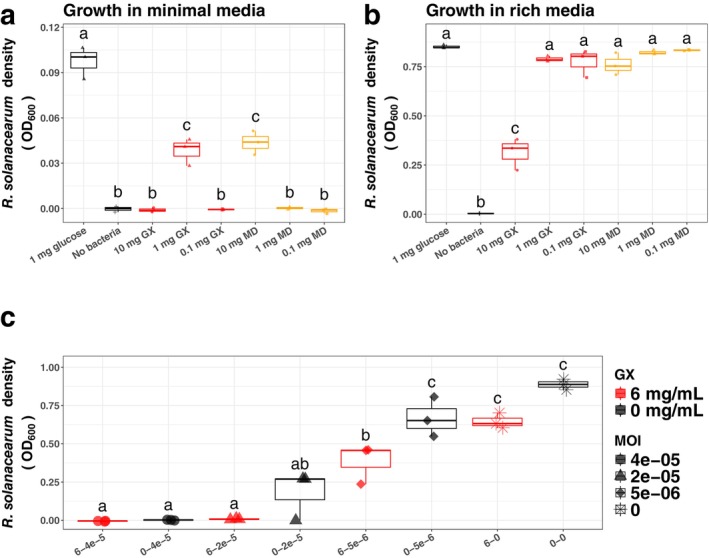
GX alone can inhibit RS growth at high concentrations and serve as a carbon source at low concentrations. (a) Both MD and GX can serve as sole carbon sources for RS in minimal media. RS was grown in BMM minimal media for 48 h with 10, 1 or 0.1 mg/mL GX or MD as the sole carbon source. 1 mg/mL glucose was included as a positive control. MD could promote RS growth at 10 mg/mL, where GX promoted growth at 1 mg/mL but did not at 10 mg/mL. (b) GX at high concentrations inhibits RS growth in rich media. RS was grown in rich CPG media for 48 h with the same added carbon sources as in (a). RS grew similarly to CPG + glucose for all concentrations of MD. GX significantly inhibited RS growth at concentrations of 10 mg/mL. In (a) and (b), different letters indicate significant differences between treatments (one‐way ANOVA, followed by Tukey post hoc analysis, *p* < 0.05). (c) GX and phage PYO4 can synergize to increase inhibition of RS. RS was grown in CPG media with or without PYO4 and GX at varying concentrations, starting from an initial RS concentration of 1 × 10^6^ CFU/mL. GX addition increased the inhibition of the phage at all MOIs except 4 × 10^−5^ (one‐way ANOVA followed by Sidak multiple comparison test of the effect of GX by MOI, *p* < 0.05).

Wood hemicelluloses extracted by pressurised hot water flow contain a complex mixture of many compounds, including sugars, complex carbohydrates and phenolic lignin‐derived compounds (Kilpeläinen et al. 2014). This mixture could not only contain potential nutrient sources for RS but could also contain compounds with direct antimicrobial activity (Xu et al. 2025; Li et al. 2023; Genin and Denny 2012). To investigate any complicating effects the excipients themselves might be having on RS growth, we tested RS growth with GX and MD in the absence of phage. To first determine if RS can use either GX or MD as sole carbon sources, we grew RS in minimal media with either glucose, GX, or MD at 10 mg/mL, 1 mg/mL and 0.1 mg/mL concentrations (Figure 4a). Relative to 1 mg/mL glucose, GX and MD supported RS growth to a much lesser degree. However, 1 mg/mL GX and 10 mg/mL MD could serve as sole carbon sources and promoted significant bacterial growth compared to minimal media with no carbon source. Intriguingly, cultures containing GX at 10 mg/mL showed no sign of growth, suggesting GX could have antimicrobial effects at higher concentrations. We then treated RS growing in rich CPG media with either GX or MD at the same concentrations (Figure 4b). While MD did not alter RS growth at any of the tested concentrations, GX reduced RS growth at 10 mg/mL, reinforcing the hypothesis that although 1 mg/mL GX can serve as a carbon source for RS, GX itself has antimicrobial activity at higher 10 mg/mL concentration. To determine if the inhibitory activity of GX could synergistically change the susceptibility of RS to phage, we grew RS with GX, PYO4, or both at a range of concentration combinations (Figure 4c). We observed some synergy between GX and phage on RS inhibition, with significantly higher inhibition observed with the addition of 6 mg/mL GX at multiple phage MOIs (multiplicity of infection). To further test if GX‐encapsulated phage had stronger inhibitory effects than non‐encapsulated phage alone, we grew RS in the presence of GX alone, GX‐encapsulated phage, or GX with unencapsulated phage at a range of concentrations (Figure S7). While GX‐encapsulated phage inhibited RS growth similarly to unencapsulated phage, GX combined with unencapsulated phage had slightly lower inhibition than phage alone or encapsulated phage, suggesting that encapsulation could alter the antibacterial activity of GX or enhance phage efficacy.

### 2.7 Both GX and MD Alone or When Encapsulated With Phage Can Reduce Bacterial Wilt Disease on Tomato

**FIGURE 5:**
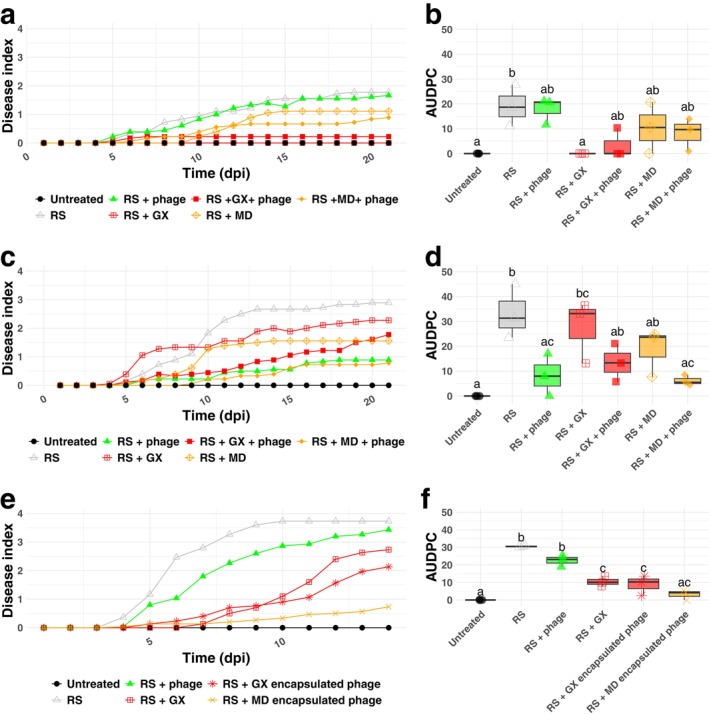
Encapsulated and unencapsulated phage, GX and MD can all reduce RS symptoms on tomato. Tomato were challenged with RS through soil drench inoculation. Disease symptoms were rated daily (a, c and e) using a disease index scale based on the percentage of wilted leaves of the plants, from 0 (no sign of disease) to 4 (plant is completely wilted). (b, d and e) AUDPC were calculated and used for statistical comparisons, with different letters indicating significant differences between treatments (one‐way ANOVA, followed by Tukey post hoc analysis, *p* < 0.05). Each point represents the average of three biological replicates, each containing six to ten individual plants. All treatments of RS, phage suspension, or excipient suspension, were applied in 10 mL volumes. We tested multiple concentrations of phage, excipient and RS. (a) and (b) excipients were applied at a concentration of 10 mg/mL and phage applied with a total of 10^5^ PFU/mL. RS was added at a concentration of 10^7^ CFU/mL, leading to a final MOI of approximately 0.01. (c) and (d) excipients were applied at a concentration of 2 mg/mL and phage applied with a total of 10^7^ PFU/mL. RS was added at a concentration of 2.5 × 10^7^ CFU/mL, leading to a final MOI of approximately 0.4. (e) and (f) phage, excipient and bacteria were added to the same concentration as in (a). Encapsulated phage were added such that the final MOI was the same as with the unencapsulated phage and the excipient concentration was approximately the same as GX without phage.

Finally, we tested the efficacy of GX, MD and non‐capsulated phages combined with GX or MD in controlling bacterial wilt disease in vivo using tomato as a model system. To begin, we treated tomato with either unencapsulated phage, GX alone, MD alone, or combinations of both excipient and unencapsulated phage in pairwise combinations. RS was added at a concentration of roughly 10^7^ CFU/mL, phage was added to a final MOI of 0.01, and excipients were added at 10 mg/mL (Figure 5a). GX alone significantly reduced the area under the disease progression curve (AUDPC), but no significant differences were observed upon the addition of non‐encapsulated phages, MD, or either combination treatment (Figure 5b). However, there was a trend towards reduced symptoms in both MD treatments and the GX + phage treatments. Overall, the level of wilting symptoms was low in this experiment. Therefore, we repeated the test using a higher dose of RS (about 2.5 × 10^7^ CFU/mL), a higher MOI for the unencapsulated phage (0.4) and lower concentrations of GX and MD (2 mg/mL). These conditions yielded overall greater levels of wilting symptoms (Figure 5c). Here, significant reductions in symptoms were observed for unencapsulated phage alone and unencapsulated phage plus MD (Figure 5d). There was also a clear trend towards reduced symptoms in unencapsulated phage plus GX or MD compared to GX and MD alone, although the differences were not significant. However, no significant differences could be observed between unencapsulated phage alone and phage plus GX or MD. Overall, these results indicated that both the phage and GX can reduce bacterial wilt symptoms, although the effects are dose dependent.

We finally tested the ability of phage encapsulated with GX or MD to reduce wilting symptoms on tomato. We inoculated tomato again with RS at 1 × 10^7^ CFU/mL and phage at an MOI of 0.01. At these concentrations, the concentration of excipient in the encapsulated phage samples was around 10 mg/mL. In the absence of any phage or excipient treatment, RS‐inoculated plants reached a disease index of 3.8, by day 14 and no wilting symptoms were observed in the absence of RS (Figure 5e). Both encapsulated phage treatments significantly reduced disease progression compared to RS‐alone treatment (Figure 5f). Further, both encapsulated phage treatments had significantly lower levels of disease compared with plants treated with unencapsulated phage. In these conditions, unencapsulated phage alone did not significantly reduce symptoms compared with RS‐alone, although there was a trend towards reduced disease. Again, GX alone significantly reduced symptom development compared to RS‐alone and unencapsulated phage, although it was not different from GX‐encapsulated phage. These results demonstrate that encapsulated phage can be used to reduce bacterial wilt symptoms on tomato and suggest that encapsulation excipient may enhance the protective efficiency of phage treatments. For GX, this might be mediated by its own ability to inhibit RS growth and reduce disease.

## 3 Discussion

While phage have great potential for use in the control of agricultural pathogens, their diversity complicates the development of standard storage and application procedures and having a similarly diverse range of storage methods and excipients can increase the number of phages available for biocontrol applications. Here, we investigated the applicability of two wood hemicelluloses to preserve and store a lytic phage of the plant pathogen *R. solanacearum* using spray drying.

Our spray drying method generally produced encapsulated phage powders with reasonable process yields (i.e., the amount of powder recovered after the spray drying process that is not lost from adherence to the apparatus walls or loss through the exhaust), a low moisture content, and generally uniform particle sizes. Only some of these aspects were influenced by spray drying temperature. There was no significant effect of spray drying temperature on the yield, but there was a trend towards increased yield in the high temperature treatments. This aligns with broader encapsulation literature that identifies thermal efficiency as critical for powder recovery (Ho, Abik, et al. 2023; Bhandari et al. 1997). The current study's results are in line with recent research on probiotic encapsulation using wood hemicelluloses, where GX and GGM microcapsules achieved process yields of 50%–58% and 35%–49%, respectively, with GX outperforming MD in most cases (Eveliina et al. 2024). Water levels are another key factor affecting microcapsule stability and activity. All three excipients exhibited low moisture levels after spray drying. While the excipient material had no statistically significant impact on final moisture content or water activity, drying temperature did significantly influence the water content, with low temperature treatments generally having higher moisture content and water activity. In all cases the observed moisture content and water activity values align with recommended thresholds for amorphous biopolymer stability (moisture content < 10%; water activity < 0.3), minimising molecular mobility and phage degradation during storage (Corcoran et al. 2004; Łopusiewicz et al. 2021). All formulations achieved water activity ≤ 0.36, well below the critical threshold (0.6) for microbial proliferation, similar to previous studies using GX and GGM as spray drying excipients (Eveliina et al. 2024; Ho et al. 2024). Excipient selection critically governs process efficiency in encapsulation systems and based on our results the hemicellulose‐based materials offer sustainable alternatives to traditional encapsulants (Eveliina et al. 2024; Ho, Abik, et al. 2023).

While all spray drying conditions produced physical particles with desirable qualities, the most important factor to consider is their ability to protect phage from degradation during the spray drying process. In almost all cases, surviving phage could be detected in the powder after spray drying, although the survival rate depended on both spray drying temperature and excipient. The reduction of titre for all three excipients was higher for the phages dried at high temperatures compared to those dried at low temperatures, indicating a sensitivity to heat that leads to phage inactivation (Malik et al. 2017). GX had the highest survival during the encapsulation process, with a phage titre reduction of only 2 and 3 log after spray drying at low and high temperatures respectively. This contrasted with GGM, which had the lowest yields after spray drying, with titers dropping between 3 and 5 log. One GGM replicate, which used an initial solution with lower phage titre, had no detectable phage after spray drying, highlighting the need for high titre starting material for the storage process. For this phage, there was a clear difference in survival between the two hemicellulose mixtures. While GX and GGM have been adapted for similar applications in the encapsulation of a variety of biological molecules, they do differ in their molecular structure and component sugars (Eveliina et al. 2024). More detailed studies could dissect any individual interactions between the hemicelluloses and phage components, and there remains the possibility that GGM could better complement other phage species. Compared to previous studies storing phages in solid formulations, our phage survival rates were generally comparable to previous results. The survival rate of phage encapsulated using lyophilization, depending on the phage itself and the excipient mixture used, varies widely. A study of RS phages found high survival rates for one phage, with around only 1 log drop in titre, but drops between 2 and 3 log for two other phages (Álvarez, Gadea‐Pallás, et al. 2022). A study of one *E. coli* infecting podovirus found widely ranging survivals based on the material used, ranging from less than 1 log drop to a 4 log drop (Dini and de Urraza 2013). Spray drying can have comparable range efficiencies, with some studies finding titre losses of only 0.2 log (Chang et al. 2019) and others as high as 2 log drops (Li, Chang, et al. 2021). While our results are generally within the yields of surviving phage observed in previous studies, it is likely that the excipient mixture and spray drying process could be further refined to increase phage survival during the process.

While the powders dried at high temperature did have lower moisture content, this did not seem to lead to higher stability over the course of the study, suggesting that the lower temperature might be preferable for future development. Instead, the storage temperature had the strongest impact on phage stability. Over the course of 20 weeks, powders dried at both low and high temperatures for all three excipients showed very little loss of phage viability when stored at 4°C. At room temperature, there was more loss of phage over time and there did not seem to be much difference between phage dried at low or high temperatures. Between the hemicellulose materials, GGM was the least stable when stored at both conditions. This is consistent with previous findings, where GX and GGM were used as excipients for yeast cells through spray drying and GGM showed significantly lower stability (Ho et al. 2024). Given that we could not accurately determine the phage titre for one of the GGM powder samples and the titre in the remaining samples was too low to be used at an equivalent MOI in in planta experiments, GGM was excluded from further analyses. While this suggests that the specific makeup of hemicelluloses used in spray drying is significant for the performance of the process, it also raises the possibility that further refining of the spray drying excipient could produce powders with higher phage density and better survival. Phage encapsulated in MD maintained the greatest stability, even at room temperature. MD has been used as encapsulating agents for many years, due to its good emulsifying properties, high glass transition temperature and high water solubility (Xiao et al. 2022). Phages encapsulated in both wood hemicelluloses and MD maintained their infectivity in vitro and phages encapsulated in GX or MD were unaffected in their ability to bind to their RS hosts. Overall, the wood hemicelluloses have great potential for use in spray drying, with GX especially providing better protection than MD during the spray drying process. However, their reduced ability to promote long‐term phage survival at room temperature compared to MD is an important limitation. More work is needed to determine if an excipient mixture could be designed to encompass both the spray drying protection of GX and the storage stability of MD.

Besides temperature, other environmental stressors can greatly limit biocontrol efficacy by killing phage before they can encounter their target host (Ignoffo and Garcia 1994; Jones et al. 2008; Enebe and Erasmus 2024). UV radiation, for example, can be a great challenge for phage survival (Ignoffo and Garcia 1994). Interestingly, we found that phages encapsulated in GX and MD had relatively high survival compared to phage suspensions in SM media. GX additionally could protect phage after resuspension, albeit at relatively high concentrations. While UV stress is generally higher for foliar‐applied phage compared to soil‐applied phage (Jones et al. 2008), as was used here, the increased UV protection could be beneficial during storage and initial application of phages onto topsoil of plants where they are exposed to UV. In the future, it would be interesting to test if hemicellulose encapsulation could protect foliar biocontrol phages from UV during the application.

Excipient materials, while important for protecting the encapsulated phage from environmental stressors, are not necessarily biologically neutral. While we found that both GX and MD alone could affect RS growth in vitro by acting as a carbon source, the interactions between GX and RS were especially complicated. While GX could act as a sole carbon source for RS growing in minimal media at 1 mg/mL, 10 mg/mL GX could effectively inhibit RS growth. This is not necessarily surprising, as plant cell walls can be rich sources for antimicrobial compounds (Ishida and Noutoshi 2022; Li et al. 2023). The complex mixture produced by the hot water extraction process includes a variety of residual products with antimicrobial activities, including lignin and other phenolic compounds (Xu et al. 2025; Li et al. 2023). Determining the mechanism of this inhibition could provide valuable insight into how encapsulation with GX could enhance and synergize with phage treatments, although it is complicated by the complex nature of the GX mixture. The lignin component is a likely source of antibacterial activity, although the mechanism of inhibition by lignin can be complex and dependent on both the structure of the lignin molecule itself and the identity of the target bacteria. Lignins are often bactericidal, adhering to bacteria cell surfaces and directly killing through cell membrane disruption, ROS concentration and limiting bacterial metabolism (Li et al. 2023). Other phenols and polyphenols can also have similar bactericidal effects (Chen et al. 2024). Even the major component of GX, the hemicellulose, could play a role in RS inhibition, as hemicellulose hydrogels have been used to concentrate antibiotic compounds and bring them in close contact with target bacteria (Arellano‐Sandoval et al. 2020; Ahmad et al. 2020). Future studies are however required to better understand the specific antimicrobial mechanisms of hemicelluloses on RS.

Despite the inhibitory activity of GX, RS can still use GX as a sole carbon source. RS can survive and scavenge for sparse nutrients in the environment during the saprophytic stage of its life cycle and has the genetic capacity to use a broad range of nutrients, including derivatives and degradation products of complex plant polymers (Perrier et al. 2018; Khokhani et al. 2017; Genin and Denny 2012). In the medical and nutrition field, complex carbohydrates including a chemically modified resistant maltodextrin are routinely used as prebiotics, being selectively used by particular taxa within the microbiome (Włodarczyk and Śliżewska 2021). Soil bacteria can also use maltodextrins as carbon sources (Schönert et al. 2006). Clearly, care must be taken to examine the effects of not only the microcapsules themselves but any potential side effects on the microbiota or plant host. In our RS bacterial wilt system, this requires rigorous testing of the effects of both the spray dried materials and excipients on the soil community.

GX alone, and to a lesser extent MD, could also reduce bacterial wilt symptoms. For GX, one likely mechanism of this improvement could be the direct antimicrobial activity of GX on RS. It is yet unclear what precisely within GX is responsible for this inhibition, but many of the residual products included in GX after the extraction process, including especially lignin, are likely biologically active (Kilpeläinen et al. 2014). We did not test the effects of GX on any other bacterial species, so it is not clear if this inhibitory activity is broad spectrum or specific to RS. If it can inhibit a wide range of organisms, further care could be necessary to ensure that GX application does not open niches within the soil for invasion by RS or other pathogens or otherwise degrade the soil microbiome diversity. However, besides inhibiting RS growth, GX could have an impact through positive interactions with the tomato host or the soil microbiome. The application of simple and complex sugars to soil can influence microbial composition and can be exploited to establish disease suppressing microbial communities (Zhou et al. 2025; Tang et al. 2020). Complex polymers, such as xylans, are favoured nutrition sources of many beneficial microorganisms living in the rhizosphere, including *Flavobacteriaceae* and *Chitinophagaceae* (Liu et al. 2018; Martin et al. 2025). Other simple carbon sources can also enrich microbial communities of disease suppressing microorganisms. Wu et al. (2015) showed that pectin application to soil enhanced the biocontrol activity of *Bacillus amyloliquefaciens* against RS in tobacco plants. Any potential influences on the native microbial community might also be affected by the phage itself. Previous studies of phage applications to combat bacterial wilt disease in greenhouse conditions showed that, besides reducing RS density in the soil, repeated applications of phage cocktails increased the overall diversity of the rhizosphere community and promoted the growth of select pathogen‐suppressing bacteria (Wang et al. 2024). These effects may, however, depend on the identity of the phage and design of the phage treatment. We recently tested the effects of various combinations of four phages, including the PYO4 phage used in this study, to suppress bacterial wilt disease and affect rhizosphere competition. The effectiveness of the disease suppression and the effects on the community composition depended both on the identities of the phage and the number of phages used in the treatment. However, in this study, phage treatment tended to reduce rhizosphere community diversity, although in some cases the abundance of disease suppressive taxa was increased upon phage treatment (Franco Ortega et al. 2024). If and how these effects are altered by the co‐application of wood hemicellulose excipients warrants further and careful consideration.

Besides affecting the soil microbial community, wood hemicelluloses could play a role by priming and activating plant defence mechanisms against phytopathogens through a DAMP‐induced immune response (Guarnizo et al. 2020; Shibuya and Minami 2001; Li et al. 2008). GX and MD polymers, either in their original structure or after microbial metabolic hydrolyzation, could trigger plant defences and prime the plant to fight infection, thus explaining the observed disease reduction. Further research could dissect the impacts of GX and MD application on the microbial composition and diversity in the rhizosphere as well as gene expression and growth responses in plant hosts.

Besides retaining lytic activity in vitro, GX and MD encapsulated phage could also reduce RS infection of tomato in the soil. As we observed in liquid culture, the effects of phage and excipients were complex, depending on the concentrations of each active compound. GX at high concentrations did produce a clear reduction in bacterial wilt symptoms. While the effects of MD were not significant, there was also a trend towards reduced symptoms when applied alone at high concentrations. Unencapsulated phage can effectively reduce RS symptoms, depending on the MOI. Remarkably, we found that, when applied at the same low MOI, encapsulated phage produced a greater reduction in symptoms compared to unencapsulated phage. While we were unable to distinguish between the effects of GX alone and the encapsulated phage at the concentrations used, these results raise the possibility that encapsulation could enhance the efficacy of phage therapy. Phage‐antibiotic synergy is already a promising and effective treatment strategy for multiple human pathogens (Li, He, et al. 2021), and co‐application of phage with an antibiotic producing *Bacillus* species was found to enhance phage efficacy in the rhizosphere and increase the fitness cost of phage resistance (Wang et al. 2017). Further experiments could dissect if GX microencapsulation could be used in a similar way, either through direct synergy due to the added antimicrobial effects of GX alone, increased stability of phage in the environment due to encapsulation, or some secondary effect on the soil environment. Rationally employing excipient materials that synergistically improve phage efficiency, provide additional probiotic activity, or directly inhibit pathogens themselves could be invaluable for improving the sustainability of phage resources for biocontrol.

Overall, our results show that encapsulation of phages in wood hemicellulose could be an effective means of implementing phage biocontrol treatments. Both GX, due to its superior yield of surviving phage and positive effects on disease reduction, and MD, due to its effective preservation of phage even at room temperature, are promising targets. GX application might also be beneficial through its direct anti‐RS activity. This technique could be further refined to improve the physical properties of the powder, scale the process for industrial application and refine the stability of the resulting powder. Further, these results should be expanded to determine if they are also effective for other phages. Previous work has shown that applying cocktails of phages can reduce the evolution of phage resistance in the rhizosphere (Wang et al. 2019). Encapsulating multiple phages separately or together or employing mixtures of excipients with complementary characteristics could further allow us to expand the efficacy of this technique. These results are a promising step in the design of practical phage biocontrol for bacterial wilt disease.

## 4 Materials and Methods

### 4.1 Strains and Culture Conditions

Spray‐dried GGM and GX powders, both obtained from sawdust via pressurised hot water extraction, were supplied by Boreal Bioproducts (Espoo, Finland) and the Natural Resources Institute Finland (Luke), respectively. Their components were reported elsewhere (Ho, Lehtonen, et al. 2023; Ho, Abik, et al. 2023). Maltodextrin (DE 6.5–19.5) was obtained from Biosynth Carbosynth (Reading, UK). Prior to use, excipient materials were sterilised using UV for a total of 1 h, stirring the powders after 30 min. RS strain UW551 (Swanson et al. 2005) was routinely cultured on Casamino acids‐Peptone‐Glucose (CPG) media [5 g/L glucose, 10 g/L peptone, 1 g/L casamino acids, 1 g/L yeast extract, 16 g/L agar] at 28°C (Hendrick and Sequeira 1984). The Autographiviridae phage PYO4 was originally isolated from Thames River water in the UK in 2019 (Franco Ortega et al. 2024). To propagate PYO4, 50 mL of CPG broth was inoculated with UW551. After 24 h of growth at 28°C, the culture was inoculated with PYO4 and incubated a further 48 h. The resulting lysate was then centrifuged at 2300 ***g*** for 10 min at 4°C to remove bacterial debris. The supernatant was collected and passed through a 0.22 μm PES filter. Filtered lysate was stored at 4°C prior to use.

### 4.2 Spray‐Dried Encapsulation of Phases

Dispersions of GGM, GX and MD with a solid content of 20% (w/w) were prepared by dispersing the respective powders to sterilised MilliQ water under stirring continuously at 500 rpm for at least 24 h to ensure complete hydration and uniform dispersion. The dispersions of GGM, GX or MD was mixed with phage solution at volume ratio 1:1, resulting in a final phage concentration of approximately 10^8^ plaque‐forming units per millilitre (PFU/mL) in the feed. The feed dispersions were subsequently stirred at 500 rpm for at least 30 min to achieve homogeneous distribution of the PYO4 phage.

Spray drying was conducted using a laboratory‐scale spray drier (B‐290, Buchi Labortechnik GmbHDE, Essen, Germany) equipped with a two‐fluid spray nozzle (0.7 mm diameter). Two inlet/outlet temperatures were used: 170°C/70°C and 105°C/50°C which represented high and low drying temperature conditions, respectively. Other spray drying conditions included a compressed air pressure of 6 bar and a flow rate of drying air of 32 m^3^/h. During spray drying, feed dispersions were gently stirred to ensure their homogeneity. Powders collected in the sample collection container were used for process yield determination and subsequent analyses. Process yield (%) is calculated as the ratio of the dry solid mass of the powder to the total dry solid mass of the feed dispersions. Table 1 summarises the sample codes of the formulations of different excipients, outlet and inlet air temperatures used in this study.

### 4.3 Physicochemical Properties of the Encapsulated Phage Powders

The methods for the physicochemical characterisation of the encapsulated phage powders were described in a previous publication (Ho, Lehtonen, et al. 2023). In brief, moisture content was determined by drying the samples in an oven at 105°C for 24 h (Memmert UNE 600, Memmert GmbH Co. KG, Schwabach, Germany) until constant weight. Water activity was measured at 25°C using a water activity meter (LabMaster‐AW, Novasina AG, Lachen, Switzerland). Particle size distribution was analysed by laser light scattering with an Aero S dry powder disperser (Mastersizer Hydro 3000 SM, Malvern Instruments Ltd., Worcestershire, UK), employing a refractive index of 1.479. Morphological examination was performed by field emission scanning electron microscopy (FESEM, S‐4800, Hitachi, Tokyo, Japan) after samples were coated with a 4 nm layer of gold/palladium (208HR, Cressington Scientific Instruments, Watford, UK) over two sputtering cycles.

### 4.4 Phage Viability and Quantification

The number of viable phages in the feed dispersions (collected before spray drying) and in the freshly prepared powders was determined to evaluate phage survival after spray drying. 100 mg of each powder was dissolved in sterile water to a final concentration of 0.1 g/mL and vortexed for 8–10 min to ensure complete suspension. Viable phage particles were enumerated by soft‐agar plaque assay as follows: overnight cultures of RS were harvested by centrifugation, resuspended in sterile water and diluted into molten soft agar (4 g/L) at 50°C to achieve an OD_600_ of 0.01–0.05 before pouring onto CPG agar plates. Ten‐fold serial dilutions of each phage suspension were prepared and spotted onto the agar overlay. Plates were incubated overnight at 28°C, after which time plaques were counted to calculate phage titre.

To evaluate the stability of the powders during storage, samples in 50 mL Falcon tubes with closed caps were stored at room temperature (22°C) and at 4°C for 25 weeks. At weekly intervals, powder samples were withdrawn and analysed for the number of viable phages using the method described above.

To evaluate the UV protection of wood hemicellulose, GX and MD phage powders were resuspended in water to a final concentration between 0.05 and 0.1 g/mL. 500 μL of these suspensions were placed in an open petri dish in a laminar flow hood. Additionally, dry GX and MD phage powders were spread in a thin layer in a petri dish. These samples were then subjected to 30 min of UV treatment from a Philips TUV30W G30T8 UV bulb (Philips, Amsterdam, Netherlands) leading to a total UV dose of roughly 22.14 kJ. Afterwards, the dry powders were resuspended to a final concentration between 0.05 and 0.1 g/mL in water, and all samples were plated on an agar overlay to determine their titre. All samples were compared to identical samples incubated at room temperature for 30 min without UV treatment.

### 4.5 In Vitro RS Inhibition Assays

RS cultures were serially diluted in liquid CPG to achieve starting concentrations of 10^3^ and 10^4^ CFU/mL. Encapsulated phage powders from 2 months post‐spray drying were suspended in CPG to a concentration of 0.1 g/mL and filtered through a 0.22 μm syringe filter. Phage titers for each encapsulated formulation, as well as for unencapsulated PYO4 phage, were determined prior to the assay. Phage suspensions were added to bacterial dilutions in 96‐well plates at a final MOI of 1. For treatments involving GGM‐encapsulated phage, the initial phage concentrations were 1 × 10^2^ PFU/mL; for GX‐ and MD‐encapsulated phage treatments, as well as unencapsulated phage controls, the initial concentrations were 1 × 10^3^ PFU/mL. Each treatment was performed in triplicate in a 96‐well plate, with a final well volume of 200 μL. Control wells included sterile CPG medium and RS cultures without phage. Plates were incubated at 28°C with shaking, and the OD₆₀₀ was measured at 0, 24, 48 and 72 h. GX‐phage synergy assays were conducted using bacterial cultures starting at 1 × 10^6^ CFU/mL and the OD₆₀₀ was measured at 24 h.

### 4.6 Phage Adsorption Assays

Samples of GX‐L and MD‐L (Table 1) stored at 4°C for 6 months were rehydrated and incubated at 28°C for 30 min both in the presence and in the absence of RS (MOI = 0.001). GGM was excluded due to a low titre at that time point. Following incubation, the suspensions were centrifuged at 10000 ***g*** for 10 min, and the resulting supernatants were sterilised through 0.22 μm PES syringe filters. Phage titers in the filtered supernatants were determined by soft‐agar plaque assay as described above. Adsorption efficiency was determined by calculating the PFU/mL obtained in the presence of bacteria as a percentage of the PFU/mL obtained in the absence of bacteria.

### 4.7 Disease Assays In Planta

RS disease assays were conducted on susceptible tomato plants (cv Moneymaker) as described in (Khokhani et al. 2018). Seeds were germinated in peat soil and grown in a 28°C growth chamber under a 12 h light/12 h dark cycle, with watering as needed; at 14 days post‐germination, seedlings were transplanted into individual 15 × 15 cm pots. At 18 days post‐germination, plants were inoculated by applying 10 mL of a 10^7^ CFU/mL RS suspension around the base of the plant, immediately followed by 10 mL of encapsulated phage suspensions (GX‐L and MD‐L) at a final concentration of 10^5^ PFU/mL (MOI = 0.01). Again, GGM was excluded due to low phage titre. Three biological replicates per treatment (each consisting of a tray containing 10 plants) were included. To assess the effect of the excipient material alone, 10 mL of sterile GX solution (0.01 g/mL) without phage was applied to a separate set of plants. Positive controls included plants inoculated with RS only and plants treated with 10^5^ PFU/mL unencapsulated PYO4 phage, while negative controls remained untreated. Disease progression was evaluated daily for 14 days using a wilt severity scale (0 = no wilt; 1 = 1%–25% leaves wilted; 2 = 26%–50%; 3 = 51%–75%; 4 = 76%–100% wilted; Tans‐kersten et al. 2001).

### 4.8 Experimental Design and Data Analysis

Data analysis was carried out with one‐way ANOVA and repeated measures two‐way ANOVA, performed with R on RStudio 2024.12.0. Differences between treatments were analysed using mixed ANOVA with R on RStudio 2024.12.0 (Schandry 2017). Plots were created using ggplot2 (Wickham 2016). Factorial ANOVA for phage survival during storage was conducted using the native Anova R package and the emmeans package for post hoc testing (Lenth and Piaskowski 2025).

## Author Contributions

**Alba M. Negroni:** methodology, visualization, investigation, writing – original draft, conceptualization, formal analysis. **Connor G. Hendrich:** conceptualization, investigation, writing – original draft, writing – review and editing, visualization, validation, formal analysis, supervision, data curation, methodology. **Minh Amin Yousefvand:** conceptualization, investigation, writing – review and editing, writing – original draft. **Ville‐Petri Friman:** conceptualization, funding acquisition, writing – review and editing, project administration, supervision.

## Funding

The research was funded by the Research Council of Finland.

## Conflicts of Interest

The authors declare no conflicts of interest.

## Supporting information


**Appendix S1:** mbt270315‐sup‐0001‐AppendixS1.docx.

## Data Availability

The data supporting these findings are available in the article and Appendix S1.
