# Correction to ‘Wood Hemicellulose‐Based Spray‐Dried Microencapsulation of a Lytic Bacteriophage Preserves Phage Viability and Improves Control of the Bacterial Wilt Pathogen *Ralstonia solanacearum*’

**DOI:** 10.1111/1751-7915.70347

**Published:** 2026-04-06

Negroni, A. M., C. G. Hendrich, M. T. Ho, A. Yousefvand, and V. P. Friman. 2026. ‘Wood Hemicellulose‐Based Spray‐Dried Microencapsulation of a Lytic Bacteriophage Preserves Phage Viability and Improves Control of the Bacterial Wilt Pathogen Ralstonia solanacearum.’ *Microbial Biotechnology* 19, no. 2: e70315. https://doi.org/10.1111/1751‐7915.70315.

The author contribution of Minh Thao Ho was removed from the approved proof prior to publication. The author contributions should read:

**Alba M. Negroni:** methodology, visualization, investigation, writing – original draft, conceptualization, formal analysis. **Connor G. Hendrich:** conceptualization, investigation, writing – original draft, writing – review and editing, visualization, validation, formal analysis, supervision, data curation, methodology. **Minh Thao Ho:** Investigation, conceptualization, methodology, writing – review and editing. **Amin Yousefvand:** conceptualization, investigation, writing – review and editing, writing – original draft. **Ville‐Petri Friman:** conceptualization, funding acquisition, writing – review and editing, project administration, supervision.

We apologize for this error.

