# Supplementary material for: Wood Hemicellulose‐Based Spray‐Dried Microencapsulation of a Lytic Bacteriophage Preserves Phage Viability and Improves Control of the Bacterial Wilt Pathogen Ralstonia solanacearum
Source: Microb Biotechnol. 2026 Feb 13;19(2):e70315. doi: 10.1111/1751-7915.70315 (PMC12904778; doi:10.1111/1751-7915.70315)
Supplement: Supplementary file 1 — Appendix S1: mbt270315‐sup‐0001‐AppendixS1.docx. [file MBT2-19-e70315-s001.docx]

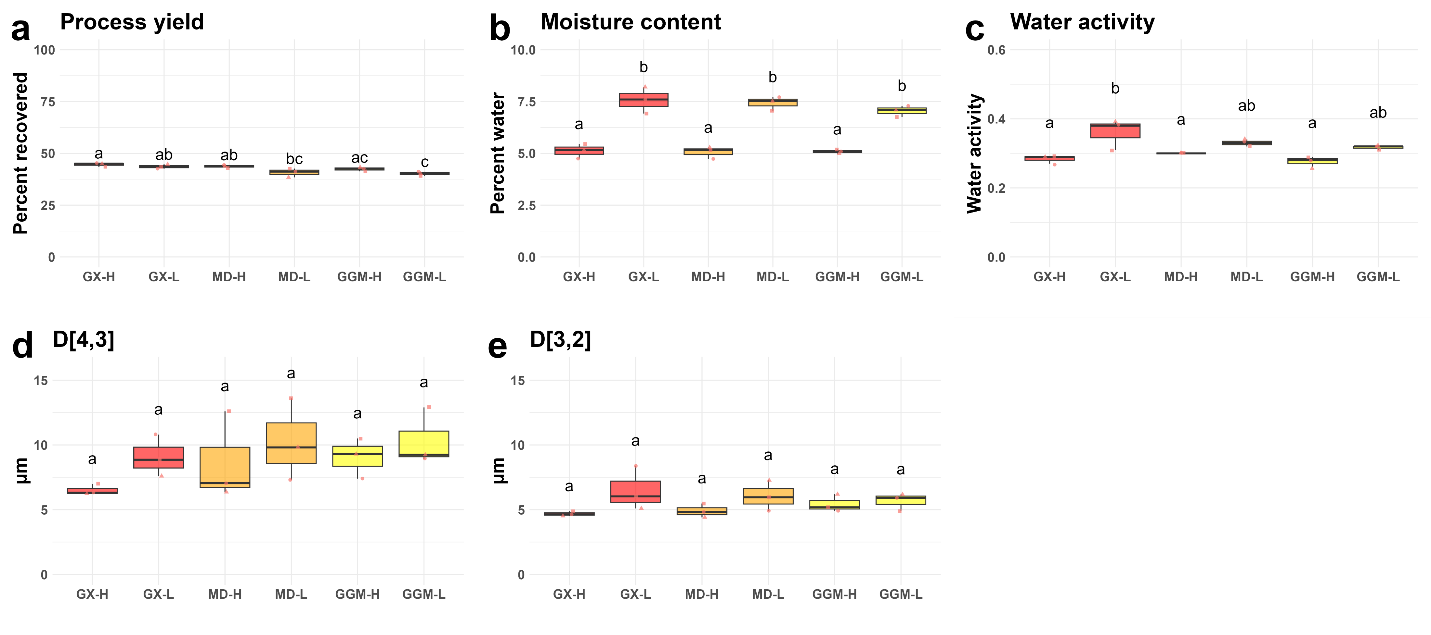


**Supplementary Figure 1. Physical characteristics of spray dried particles.** After three spray drying runs with either GX, MD, or GGM as excipients and using drying temperatures of either 170/70°C (H) and 105/50°C (L), the physical characteristics of the particles were measured. These included process yield (a), moisture content (b), water activity (c), particle volume-weighted mean diameter (d), and surface area-weighted mean diameter (e). Differences between treatments were estimated using one-way ANOVA with Tukey post-hoc analysis (different letters indicate significant differences, *p < 0.05*). Figure values are included in Table 1.


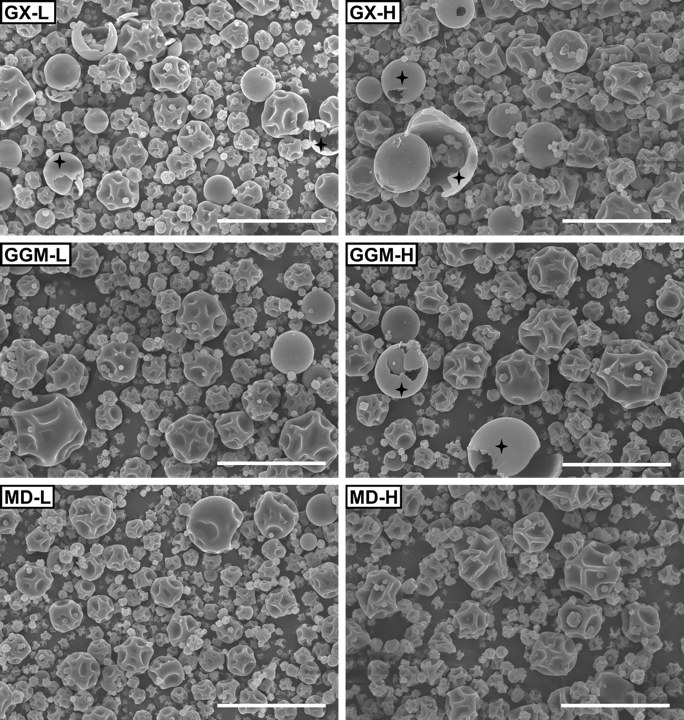


**Supplementary Figure 2. Spray drying produces phage-enclosing particles of high quality.** SEM images of PYO4 microcapsule powders produced from different types of wall materials (GX, GGM and MD) dried at either low (L) or high (H) temperatures. Scale bars indicate 20 µm. Stars indicate examples broken or ruptured particles.


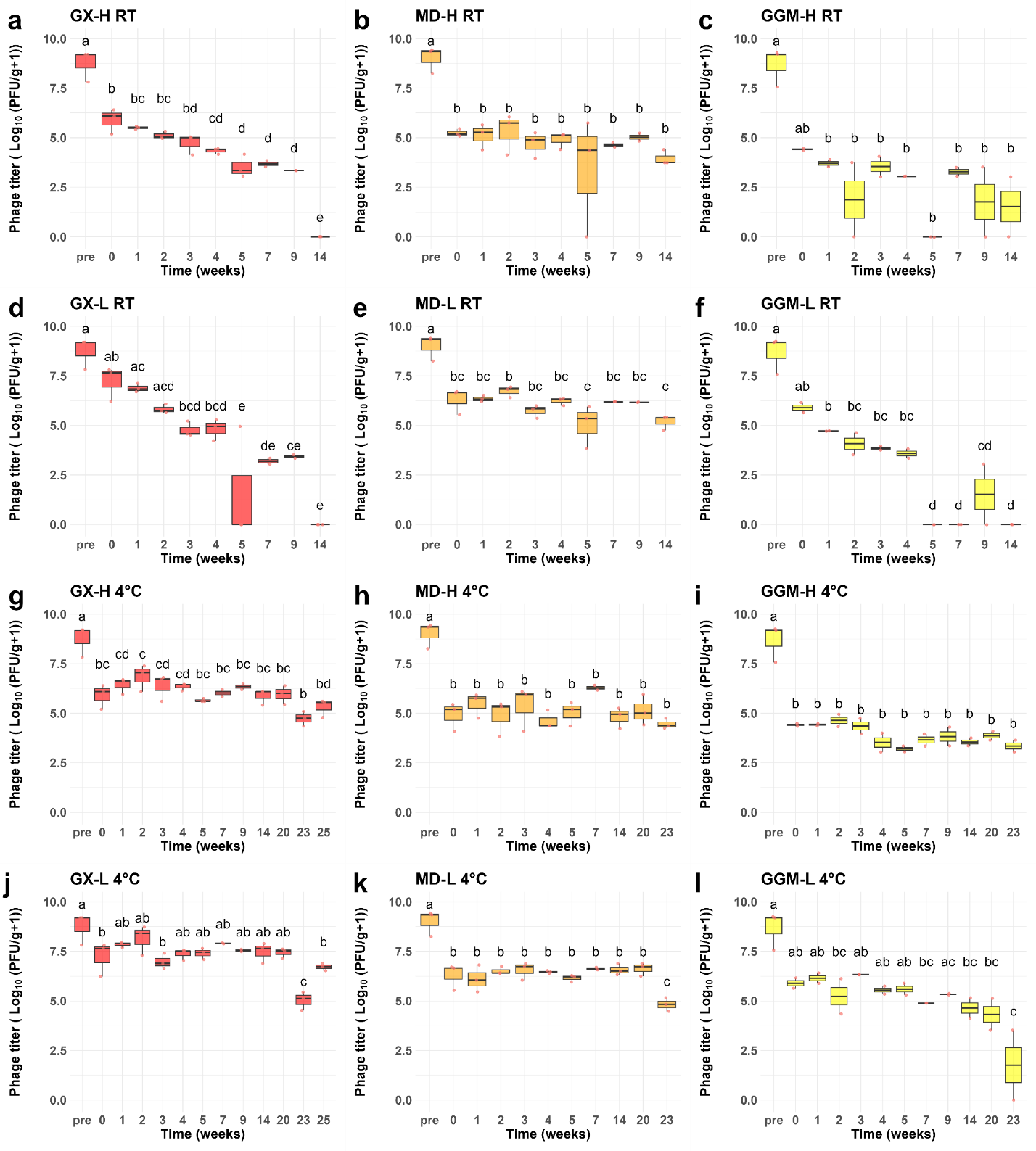


**Supplementary Figure 3. The stability of encapsulated phage was primarily affected by storage temperature.** Phage titers were measured either prior to spray drying (pre) or weekly for the following 14-25 weeks. Phages were encapsulated with: GX (glucuronoxylans) (a, d, g, j), MD (maltodextrin) (b, e, h, k) and GGM (galactoglucomannans) (c, f, i, l). Spray drying was conducted at two different temperatures: high temperature (170/70°C) (a-c, g-i) and low temperature (105/50°C) (d-f, j-l). The resulting powders were stored at room temperature (RT) (a-f) or at 4°C (g-l). Each experiment was conducted in triplicate. The limit of detection in these assays was equal to a log10(PFU/mL+1) of 3.05. Statistically significant differences between time points are indicated by different letters (one-way ANOVA, followed by Tukey post-hoc analysis, *p < 0.05*).


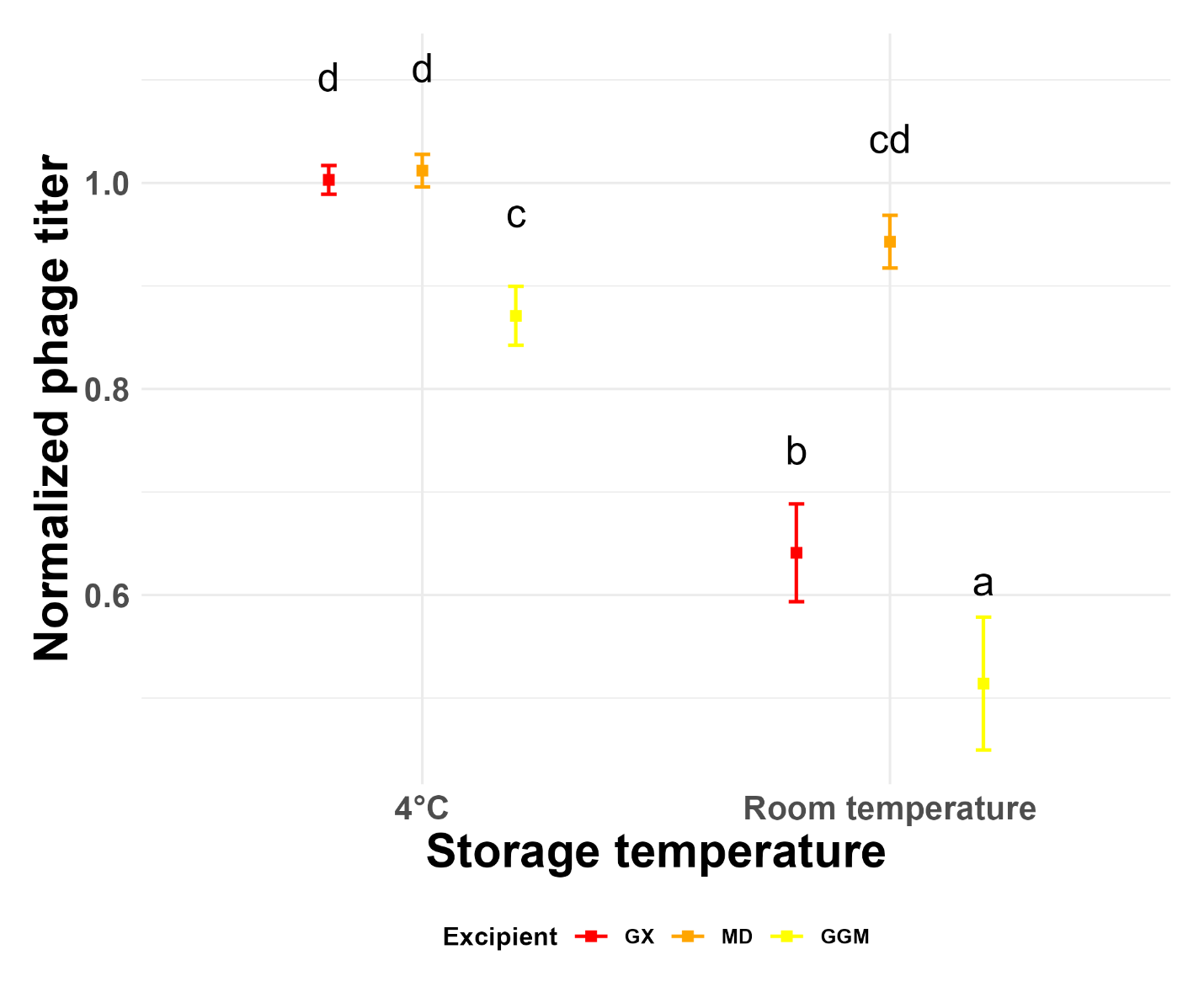


**Supplementary Figure 4. The survival of encapsulated phage depended on the excipient material and storage temperature.** Phage titers were log-transformed and normalized to the starting titer for each condition. Values represent normalized phage titers averaged over the time course and averaged by drying temperature. Statistically differences are indicated by differing letters (Sidak post-hoc analysis, *p < 0.05*).


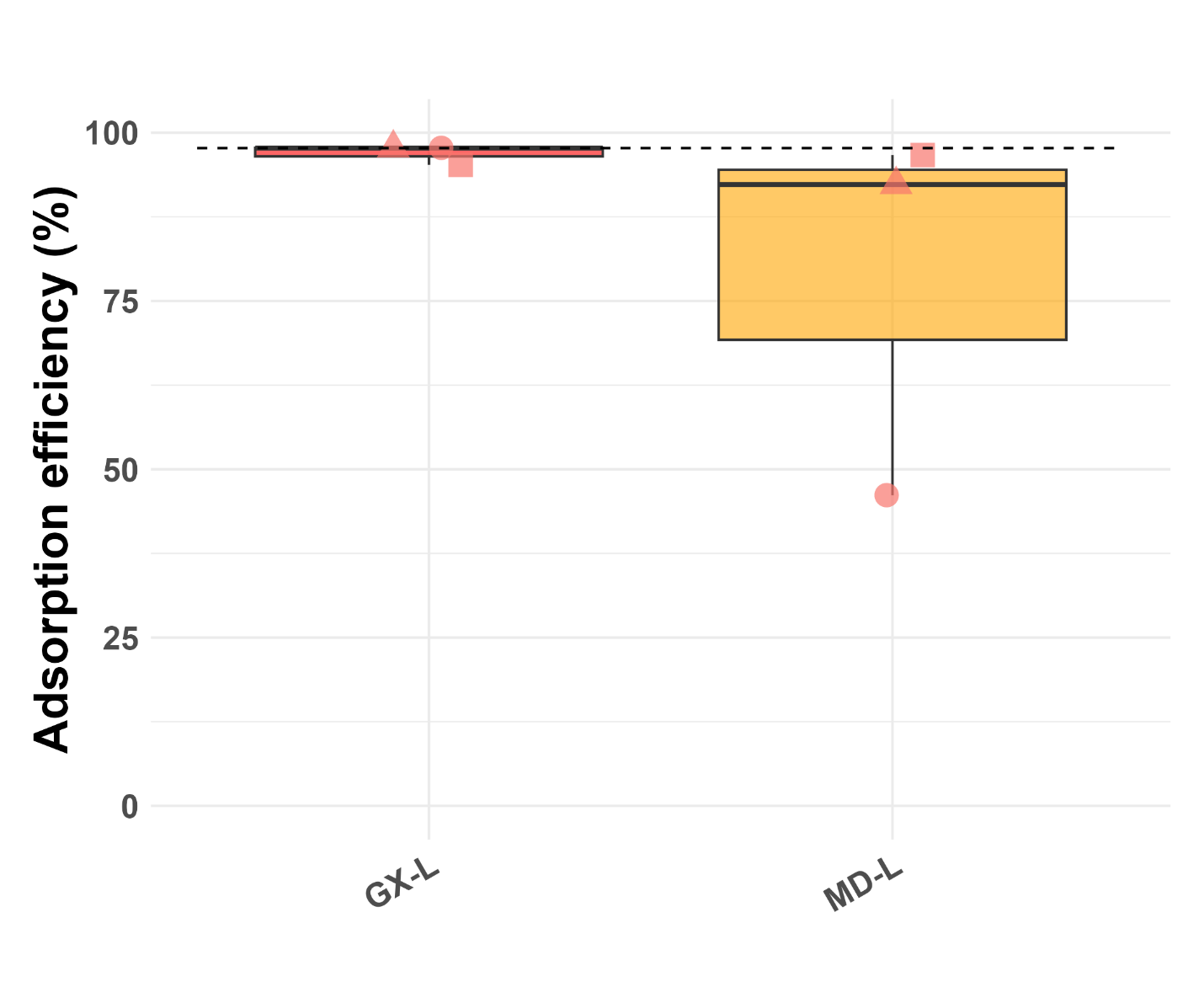


**Supplementary Figure 5. Encapsulation with GX or MD at low temperature doesn’t affect phage binding efficiency.** The binding efficiency was estimated by incubating phage samples with RS for 30 minutes, followed by centrifugation and filtration to remove bacterial cells and attached phage. The phage titer was measured in the resulting filtered supernatant and compared to samples incubated without bacteria. Neither GX nor MD encapsulated phage had a binding efficiency significantly different than unencapsulated phage (one-sample t-test, *p > 0.05*). Dotted line indicates the binding efficiency of unencapsulated phage.

**
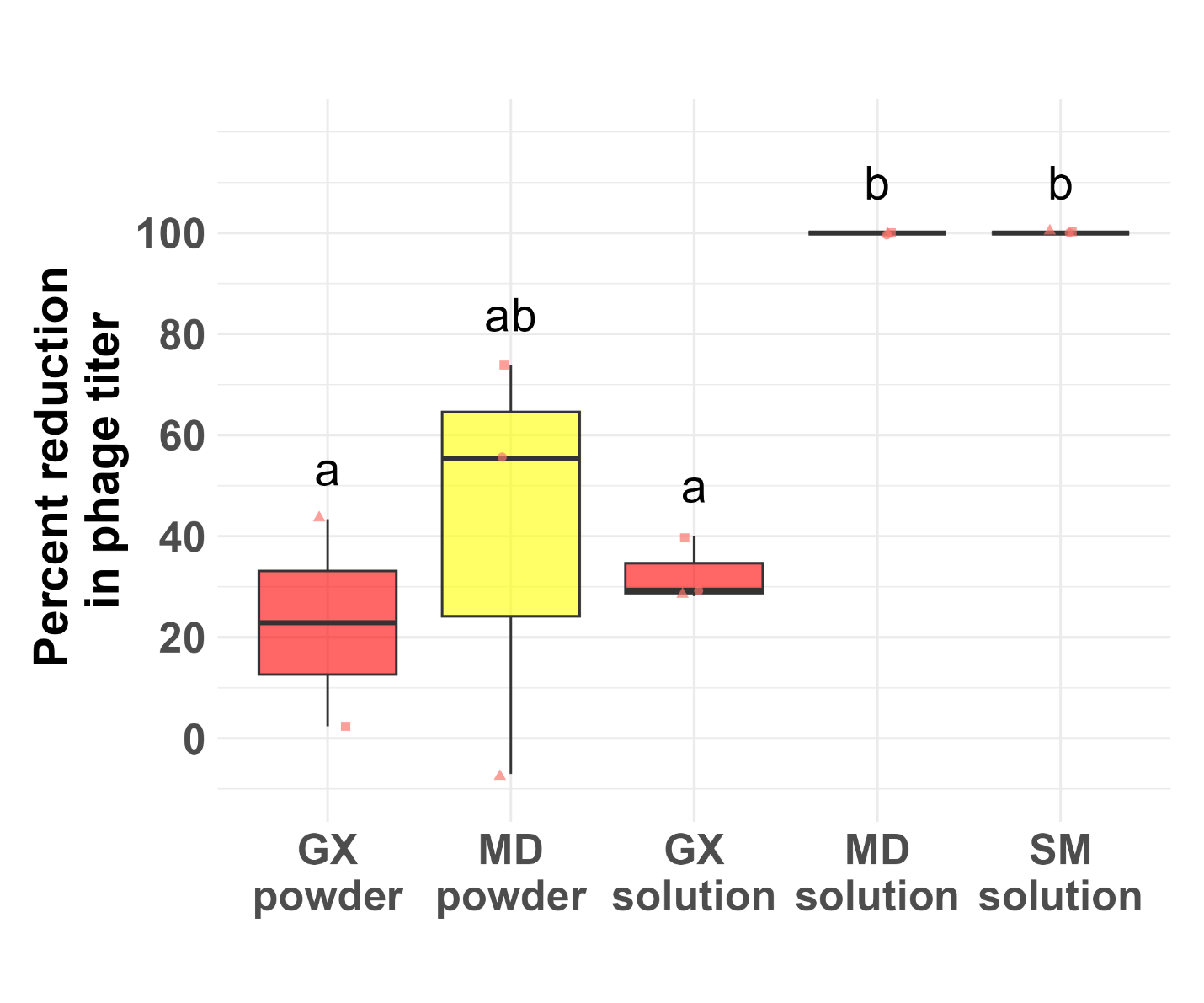
Supplementary Figure 6. Encapsulation of phage provides protection from UV damage.** GX and MD were exposed to roughly 22.14 kJ of UV radiation in 30 min either in solid powder form or after being resuspended in water to a final concentration of 0.05-0.1 g/mL excipient. Different letters indicate significant differences between treatments (one-way ANOVA, followed by Tukey post-hoc analysis, *p < 0.05*).


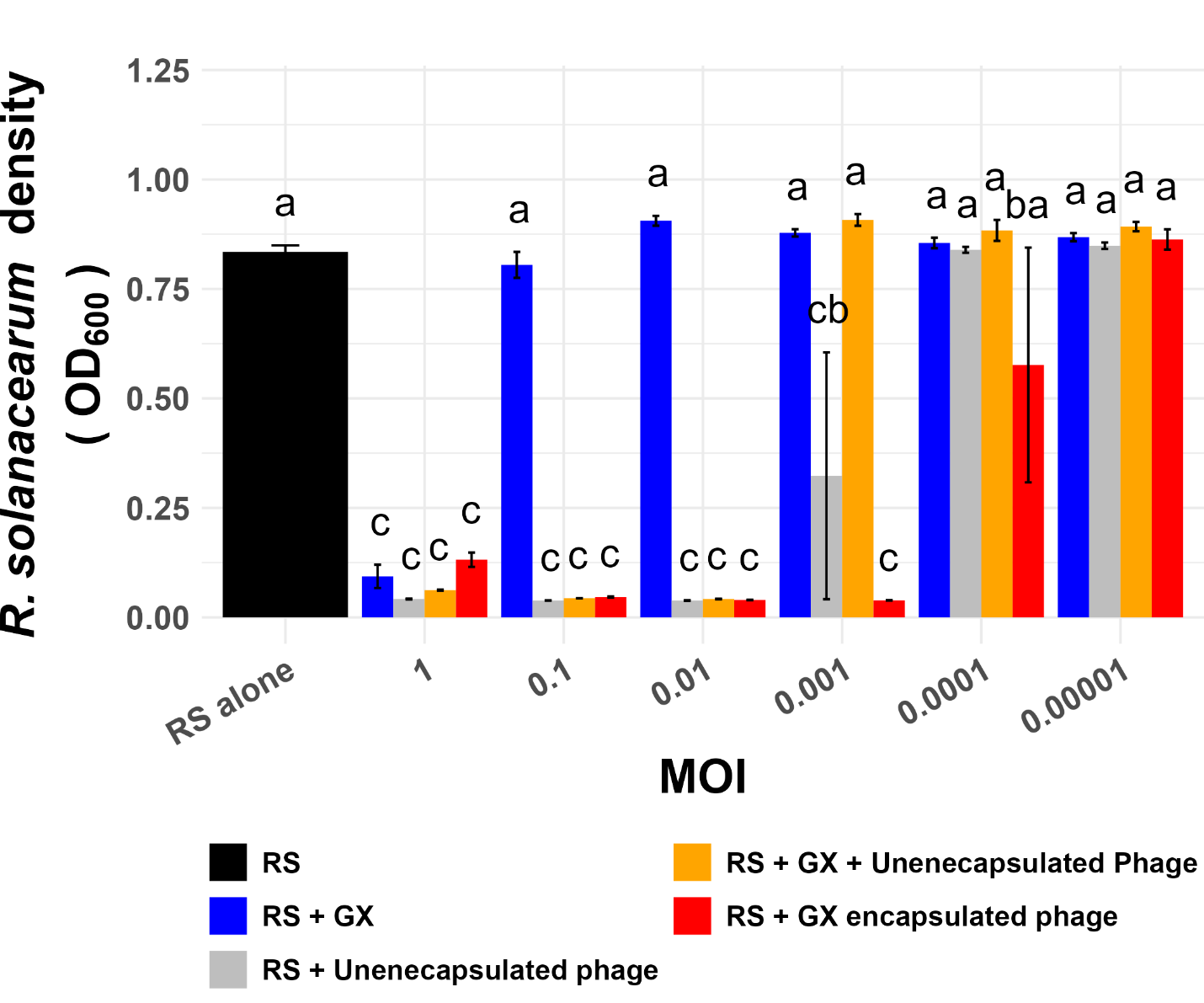


**Supplementary Figure 7. Encapsulation with GX does not affect synergy between phage PYO4 and GX.** RS was grown in rich media for 24 hours with or without phage or GX hemicellulose. Phage and hemicellulose were added either separately, together, or with phage encapsulated by GX. Phage treatments were added in a series of dilutions to produce a range of starting MOIs from 1 to 10-5. GX powder, either alone or with phage, was added to a starting concentration of roughly 0.01 g/mL and diluted in the same pattern as the phage. GX alone only inhibited RS growth at the highest concentration. The encapsulation of phage in GX did not change the activity of the phage compared to unencapsulated phage and the addition of GX to unencapsulated phage did not increase its activity. Different letters indicate significant differences between treatments (one-way ANOVA, followed by Tukey post-hoc analysis, *p < 0.05*).

**Supplementary Table 1.** Effects of excipient, storage temperature, and drying temperature on long-term survival of encapsulated phage. Factorial ANOVA and postdoc analysis was conducted using the R packages Anova and emmeans (Lenth and Piaskowski 2025).

| Anova Table (Type II tests) |  |  |  |  |  |
| --- | --- | --- | --- | --- | --- |
| Factor | Sum Sq | Df | F value | Pr(>F) | Significance |
| Week | 5.1032 | 11 | 12.874 | < 2.2e-16 | * |
| Excipient | 3.295 | 2 | 45.7183 | < 2.2e-16 | * |
| DryingTemp | 0.1386 | 1 | 3.8463 | 0.05084 |  |
| StorageTemp | 5.4134 | 1 | 150.223 | < 2.2e-16 | * |
| Excipient:DryingTemp | 0.0383 | 2 | 0.5315 | 0.58828 |  |
| Excipient:StorageTemp | 1.5254 | 2 | 21.1652 | 2.72E-09 | * |
| DryingTemp:StorageTemp | 0.0463 | 1 | 1.2845 | 0.25802 |  |
| Excipient:DryingTemp:StorageTemp | 0.0816 | 2 | 1.1326 | 0.32364 |  |
| Residuals | 10.1981 | 283 |  |  |  |
